# Association Between Antimicrobial Prophylaxis With Double-Dose Cefuroxime and Surgical Site Infections in Patients Weighing 80 kg or More

**DOI:** 10.1001/jamanetworkopen.2021.38926

**Published:** 2021-12-15

**Authors:** Rami Sommerstein, Andrew Atkinson, Stefan P. Kuster, Danielle Vuichard-Gysin, Stephan Harbarth, Nicolas Troillet, Andreas F. Widmer

**Affiliations:** 1 Department of Infectious Diseases, Bern University Hospital, University of Bern, Bern, Switzerland; 2 Swissnoso, the National Center for Infection Control, Bern, Switzerland; 3 Department of Health Sciences and Medicine, University of Lucerne, Lucerne, Switzerland; 4 University of Zurich, Zurich, Switzerland; 5 Infectious Diseases, Cantonal Hospital Thurgau, Switzerland; 6 Infection Control Program, Geneva University Hospitals and Faculty of Medicine, Geneva, Switzerland; 7 Service of Infectious Diseases, Central Institute, Valais Hospitals, Sion, Switzerland; 8 Department of Infectious Diseases, University Hospital Basel, Basel, Switzerland

## Abstract

**Importance:**

Many guidelines recommend a weight-adopted dose increase of cefuroxime for surgical antimicrobial prophylaxis (SAP). However, the evidence that this approach is associated with lower rates of surgical site infection (SSI) is limited.

**Objective:**

To assess whether double-dose cefuroxime SAP was associated with a decreased SSI rate in patients weighing at least 80 kg.

**Design, Setting, and Participants:**

This cohort study included adult patients (>18 years) weighing at least 80 kg who underwent 9 major surgical procedures with a cefuroxime SAP administration from the Swissnoso SSI surveillance system between January 2015 and December 2019 at 142 Swiss hospitals. The follow-up was 30 days for all surgical procedures and 1 year for implant-related operations.

**Exposures:**

Cefuroxime SAP dose (1.5 vs 3.0 g).

**Main Outcomes and Measures:**

Overall SSI. A mixed-effects logistic regression adjusted for institutional, epidemiological, and perioperative variables was applied. Results were stratified by weight categories as well as by wound contamination classes.

**Results:**

Of 41 076 eligible patients, 37 640 were included, with 22 625 (60.1%) men and a median (IQR) age of 61.9 (49.9-71.1) years. The outcome SSI was met by 1203 patients (3.2%). Double-dose cefuroxime was administered to 13 246 patients (35.2%) and was not significantly associated with a lower SSI rate (adjusted odds ratio [aOR], 0.89; 95% CI, 0.78-1.02; *P* = .10). After stratification by weight category, double-dose SAP vs single-dose SAP was associated with lower SSI rates among 16 605 patients weighing at least 80 to less than 90 kg (aOR, 0.76; 95% CI, 0.61-0.97; *P* = .02) but not in the other weight categories (≥90 to <100 kg, 10 342 patients: aOR, 1.12; 95% CI, 0.87-1.47; *P* = .37; ≥100 to <120 kg, 8099 patients: aOR, 0.99; 95% CI, 0.76-1.30; *P* = .96; ≥120 kg, 2594 patients: aOR, 0.65; 95% CI, 0.42-1.04; *P* = .06). After stratification by contamination class, double-dose SAP was associated with lower SSI rates among 1946 patients with contaminated wounds (aOR, 0.49; 95% CI, 0.30-0.84; *P* = .008) but not those with clean wounds (25 680 patients; aOR, 0.92; 95% CI, 0.76-1.12; *P* = .44) or clean-contaminated wounds (10 014 patients; aOR, 0.90; 95% CI, 0.73-1.12; *P* = .37) compared with a single dose.

**Conclusions and Relevance:**

In this study, double-dose SAP with cefuroxime for patients weighing at least 80 kg was not consistently associated with a lower SSI rate.

## Key Points

**Question:**

Is double-dose cefuroxime surgical antimicrobial prophylaxis associated with a lower surgical site infection rate in patients weighing at least 80 kg?

**Findings:**

In this cohort study of 37 640 patients who underwent 9 major surgical procedures, there was no significant overall association between single-dose vs double-dose cefuroxime and the outcome of surgical site infection.

**Meaning:**

These findings suggest that double-dose cefuroxime prophylaxis for patients weighing at least 80 kg may not be associated with a lower surgical site infection rate.

## Teaser

This cohort study assesses whether double-dose cefuroxime surgical antimicrobial prophylaxis was associated with the rate of surgical site infection in patients weighing at least 80 kg.

## Introduction

Surgical site infections (SSIs) account for approximately 20% of all health care–associated infections^1,2^ and have a major impact on morbidity and mortality.^3,4^ Several national and international guidelines provide evidenced-based measures to reduce SSI risk. Surgical antimicrobial prophylaxis (SAP) administration, its correct timing, and redosing have been identified as critical items for SSI prevention.^4,5,6,7,8^

While weight-adapted application of antimicrobial agents has been implemented in some infectious disease areas,^9,10^ this practice has not been widely implemented for SSI prevention.^4,5,6,7,8^ Currently, double-dose SAP administration has been shown to reduce SSI for patients weighing at least 120 kg, but all studies had sample sizes of less than 200 patients.^11,12,13,14,15^ In line with these findings, preliminary data suggest a role of double-dose SAP in reducing the SSI rate in patients who weigh at least 80 kg.^16^ Nevertheless, in most guidelines for SSI prevention, the issue of weight-adjusted SAP dosing is still considered unresolved.^4,5,6,7,8^

Even after the introduction of a nationwide SSI surveillance program, the Swiss SSI rate remained at an elevated level compared with results from other national surveillance programs.^17^ To further decrease the rate, Swissnoso, the national center for infection control, issued national guidelines in 2015 advocating the optional increase of the SAP standard dose for patients weighing at least 80 kg as part of interventions aiming at decreasing SSI rates.^7^ The aim of this study was to evaluate the association of the introduction of this recommendation in 2015 with SSI rates in Switzerland among patients weighing at least 80 kg, based on data from the Swiss nationwide surveillance program.

## Methods

SSI surveillance by Swissnoso is mandated by Swiss health care policies and is considered a quality improvement project. All patients were informed about their automatic inclusion in SSI surveillance on admission and given the opportunity to opt out. Summary results of the SSI incidences are published yearly.^18^ The Bernese Cantonal human subjects committee approved risk factors analyses within the SSI surveillance database. This study follows the Strengthening the Reporting of Observational Studies in Epidemiology (STROBE) reporting guideline.

### Study Design and Setting

This is a multicenter cohort study of prospectively collected data from the Swiss national SSI surveillance program.^17,19^ We included data from 142 health care institutions in Switzerland between January 2015 and December 2019. Each participating hospital records surveillance data on a minimum of 3 different intervention types during a selected period and then includes all patients.^17^ Patients can opt out, but this is a rare exception (<1%). The surveillance includes data collection at discharge as well as rigorous postdischarge surveillance 30 days after the intervention, with additional medical record review in case of suspected infection.^17^ For implant surgery, a second follow-up occurs after 1 year. All patients were contacted at least 5 times before being considered lost to follow-up. The overall follow-up for routine postdischarge surveillance was greater than 91%.^17^ Data were then entered in the national database. Staff members of the Swissnoso SSI surveillance team periodically performed on-site audits to check data quality, as published elsewhere.^17,19,20^

### Participants

Inclusion criteria were (1) participation in the surveillance program, (2) undergoing 1 of the 9 most frequent surgical interventions (hernia repair, knee or hip implant, cardiac surgery, laminectomy, colon surgery, cholecystectomy, cesarean delivery, and gastric bypass), (3) the procedure taking place between 2015 and 2019, (4) documented weight at the time of surgery of at least 80 kg, (5) being older than 18 years, and (6) a cefuroxime (with or without metronidazole) SAP administration of 1.5 or 3.0 g in the 120 minutes before incision. Exclusion criteria were patients with preexisting infections (ie, wound contamination class IV), missing data on SAP, and patients for whom no complete follow-up was available (Figure).

**Figure.**
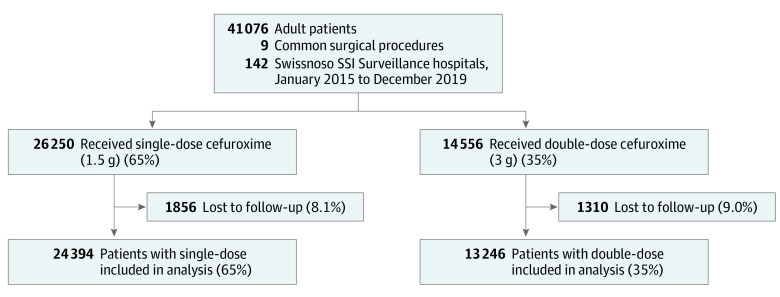
Flowchart of Patient Inclusion SSI indicates surgical site infection.

### Variables, Outcomes, and Data Sources

The primary outcome was any SSI (superficial or deep incisional infection and/or organ space infection) at 30 days and/or 1 year. Covariables included age; body mass index (BMI; calculated as weight in kilograms divided by height in meters squared); American Society of Anesthesiologists (ASA) score; wound contamination class: clean (class I), clean-contaminated (class II), or contaminated (class III); year of surgery; emergency procedure; time from SAP administration to incision (per 30 minutes); procedure duration longer than standard time; and hospital bed-size. The decision of single-dose vs double-dose SAP was in many cases decided at the level of the institution. In some institutions, however, this was also at the discretion of the surgeon and/or anesthesiologist in charge.

SSI cases were defined as patients with SSI according to US Centers for Disease Control and Prevention (CDC) definitions.^21^ Surveillance staff reviewed all patient data, and those patients with a suspected SSI were crosschecked by a dedicated physician. All supervising physicians—most board-certified in infectious diseases—had attended a training course on SSI surveillance.

Data were electronically entered into a centralized database. Type of SSI (ie, superficial incisional, deep incisional, or organ space) was recorded as well as the pathogen (if available). Primary data were obtained from the patient medical records and telephone interviews with patients. The data source for the variables was the Swissnoso SSI surveillance program.

To analyze the consequences of preoperative comorbidity, ASA scores were grouped into low (1-2) and high score (3-5). Regarding bed size, hospitals were grouped into those with fewer than 200 beds, 200 to 500 beds, and more than 500 beds.

### Statistical Analysis

To investigate differences in terms of baseline characteristics for those with single- and double-dose SAP, we used the χ^2^ or Wilcoxon tests for categorical and continuous data, respectively. We then calculated the SSI outcome for the individual interventions by single vs double SAP dosing. To determine the association between SAP dosing and SSI, we fitted covariate-adjusted, multilevel logistic regression models with clustering at the intervention level (random intercept).

Two stratified analyses, adjusted for the covariables, were performed for weight group and contamination class. A subgroup analysis excluded surgeries with a 1-year follow-up period. End point missingness resulting from patients being lost to follow-up was investigated by comparing the characteristics of the included cases with those patients lost to follow-up to determine whether there were systematic differences between the 2 groups. A 2-tailed *P* < .05 was considered statistically significant throughout. All statistics were performed in R version 4.0.2 (R Project for Statistical Computing).

## Results

We included 37 640 patients, with 22 625 (60.1%) men and a median (IQR) age of 61.9 (49.9-71.1) years. SAP was administered as single dose in 24 394 patients (64.8%) and double dose in 13 246 patients (35.2%) (Figure). The detailed baseline patient and procedural characteristics stratified by SAP dosing are shown in Table 1. Patients from higher weight groups, those with higher ASA scores, and those receiving care at larger hospitals were more likely assigned to the double-dose SAP group. Also, an increasing number of double-dose SAP was given throughout the study period (Table 1).

**Table 1.** Baseline Participant and Procedural Characteristics

Characteristic	Patients, No. (%)		*P* value
	Single dose (1.5 g) (n = 24 394)	Double dose (3.0 g) (n = 13 246)	
Age, median (IQR)	62.2 (49.7 to 71.2)	61.3 (50.1 to 70.7)	.03
Sex			
Male	13 993 (57.4)	8632 (65.2)	<.001
Female	10 401 (42.6)	4614 (34.8)	<.001
BMI, median (IQR)^a^	30.8 (28.1 to 34.3)	31.6 (28.3 to 36.3)	<.001
ASA scores			
1-2	16 806 (68.9)	7403 (55.9)	<.001
3-5	7477 (30.7)	5769 (43.6)	
NA	111 (0.5)	74 (0.6)	
Intervention type			
Total knee prosthesis	6606 (27.1)	2112 (15.9)	<.001
Total hip prosthesis	6222 (25.5)	2751 (20.8)	
Cardiac surgery	1045 (4.3)	2484 (18.8)	
Colon surgery	2226 (9.1)	1040 (7.9)	
Hernia repair	1879 (7.7)	873 (6.6)	
Cesarean delivery	2818 (11.6)	220 (1.7)	
Cholecystectomy	1574 (6.5)	839 (6.3)	
Laminectomy	872 (3.6)	968 (7.3)	
Gastric bypass surgery	1152 (4.7)	1959 (14.8)	
Wound contamination class			
I, clean	16 543 (67.8)	9137 (69.0)	<.001
II, clean-contaminated	6365 (26.1)	3649 (27.5)	
III, contaminated	1486 (6.1)	460 (3.5)	
Elective surgery	21 781 (89.3)	11 949 (90.2)	.006
SAP administration prior to incision, median (IQR), min	−38 (−50 to −25)	−39 (−50 to −28)	<.001
Surgery exceeding standard time	4657 (19.1)	1936 (14.6)	<.001
Year			
2015	1655 (6.8)	366 (2.8)	<.001
2016	7517 (30.8)	2484 (18.8)	
2017	7592 (31.1)	4200 (31.7)	
2018	5666 (23.2)	4514 (34.1)	
2019	1964 (8.1)	1682 (12.7)	
Hospital size, beds			
<200	15 411 (63.2)	6966 (52.6)	<.001
200-499	7453 (30.6)	3367 (25.4)	
≥500	1530 (6.3)	2913 (22.0)	

Abbreviations: BMI, body mass index (calculated as weight in kilograms divided by height in meters squared); NA, not available; SAP, surgical antimicrobial prophylaxis.

^a^ Data missing for 2125 patients in the single-dose group (8.7%) and 64 in the double-dose group (0.4%).

The overall rate of SSI was 3.2% (1209 patients), with 747 SSIs (3.1%) occurring in the single-dose group, and 462 (3.5%) in the double-dose group (*P* = .76). There were no differences in the crude SSI rates between the 2 groups, stratified for the individual interventions (Table 2). In the adjusted multilevel model, the double SAP dose was not significantly associated with a decreased SSI rate (adjusted odds ratio [aOR], 0.89; 95% CI, 0.78-1.02; *P* = .10). Covariables independently associated with a higher SSI risk were BMI (aOR per 1-unit increase, 1.05; 95% CI, 1.04-1.07; *P* < .001), ASA score of 3 to 5 (compared with ASA score of 1-2; aOR, 1.48; 95% CI, 1.27-1.72; *P* < .001), hospital with 200 to 499 beds (compared with <200 beds: aOR, 1.24; 95% CI, 1.07-1.43; *P* = .004), and procedures longer than standard operation time (aOR, 1.55; 95% CI, 1.35-1.78; *P* < .001). In contrast, elective surgery (aOR, 0.76; 95% CI, 0.63-0.92; *P* < .001) was significantly associated with a decreased SSI risk (Table 3).

**Table 2.** Crude Rate of SSIs, by Surgical Procedure and Cefuroxime Antimicrobial Prophylaxis Dosing

Procedure type	Patients, No.		Patients with SSI											
	1.5 g	3.0 g	Overall			Superficial			Deep wound			Organ space		
			No. (%)		*P* value	No. (%)		*P* value	No. (%)		*P* value	No. (%)		*P* value
			1.5 g	3.0 g		1.5 g	3.0 g		1.5 g	3.0 g		1.5 g	3.0 g	
Hernia repair	1879	873	15 (0.8)	7 (0.8)	>.99	10 (0.5)	5 (0.6)	>.99	2 (0.1)	2 (0.2)	.80	3 (0.2)	0	.58
Total hip prosthesis	6222	2751	118 (1.9)	56 (2.0)	.72	22 (0.4)	9 (0.3)	>.99	14 (0.2)	5 (0.2)	.87	82 (1.3)	40 (1.5)	.68
Total knee prosthesis	6606	2112	80 (1.2)	27 (1.3)	.90	22 (0.3)	9 (0.4)	.68	9 (0.1)	2 (0.1)	.91	49 (0.7)	16 (0.8)	>.99
Cesarean delivery	2818	220	86 (3.1)	5 (2.3)	.65	50 (1.8)	2 (0.9)	.50	10 (0.4)	2 (0.9)	.48	26 (0.9)	1 (0.5)	.73
Cardiac surgery	1045	2484	59 (5.6)	153 (6.2)	.61	13 (1.2)	49 (2.0)	.17	20 (1.9)	49 (2.0)	>.99	25 (2.4)	53 (2.1)	.73
Cholecystectomy	1574	839	22 (1.4)	15 (1.8)	.57	9 (0.6)	7 (0.8)	.62	2 (0.1)	1 (0.1)	>.99	11 (0.7)	7 (0.8)	.91
Laminectomy	872	968	13 (1.5)	10 (1.0)	.50	5 (0.6)	5 (0.5)	>.99	1 (0.1)	2 (0.2)	>.99	7 (0.8)	3 (0.3)	.26
Colon surgery	2226	1040	319 (14.3)	140 (13.5)	.54	88 (4.0)	40 (3.8)	.96	31 (1.4)	13 (1.2)	.87	200 (9.0)	87 (8.4)	.61
Gastric bypass surgery	1152	1959	35 (3.0)	49 (2.5)	.44	15 (1.3)	14 (0.7)	.15	3 (0.3)	3 (0.2)	.81	17 (1.5)	32 (1.6)	.85
Overall	24 394	13 246	747 (3.1)	462 (3.5)	.03	234 (1.0)	140 (1.1)	.39	92 (0.4)	79 (0.6)	.003	420 (1.7)	239 (1.8)	.59

Abbreviation: SSI, surgical site infection.

**Table 3.** Fully Adjusted Mixed-Effects Logistic Regression Models With Surgical Site Infection as the Dependent Variable^a^

Variable	aOR (95% CI)	*P* value
Cefuroxime dose		
Single	1 [Reference]	NA
Double	0.89 (0.78-1.02)	.10
BMI (per unit)	1.05 (1.04-1.07)	<.001
Age (per year)	1.00 (1.00-1.01)	.63
Sex		
Female	1 [Reference]	NA
Male	1.16 (0.99-1.35)	.06
ASA score		
1-2	1 [Reference]	NA
3-5	1.48 (1.27-1.72)	<.001
Wound contamination class		
Clean	1 [Reference]	NA
Clean-contaminated	0.76 (0.31-1.83)	.54
Contaminated	1.07 (0.44-2.60)	.88
Elective surgery		
No	1 [Reference]	NA
Yes	0.76 (0.63-0.92)	.004
Timing of SAP before incision (per 30 min)	0.92 (0.84-1.00)	.06
Duration exceeding standard time		
No	1 [Reference]	NA
Yes	1.55 (1.35-1.78)	<.001
Year		
2015	1 [Reference]	NA
2016	1.07 (0.79-1.44)	.68
2017	1.18 (0.88-1.59)	.28
2018	1.25 (0.92-1.69)	.15
2019	1.21 (0.86-1.70)	.27
Hospital size, beds		
<200	1 [Reference]	NA
200-499	1.24 (1.07-1.43)	.004
≥500	1.12 (0.92-1.35)	.26

Abbreviations: aOR, adjusted odds ratio; ASA, American Society of Anesthesiologists; BMI, body mass index (calculated as weight in kilograms divided by height in meters squared); NA, not applicable; SAP, surgical antimicrobial prophylaxis.

^a^ Procedure type was added as a random effect. Only complete cases (ie, 35 268 of 37 640 [93.7%]) included.

Given that we detected significant interactions between double-dose SAP and weight class as well as double-dose SAP and contamination class, we proceeded with stratified analyses. First, we stratified for weight categories. Among the 16 605 patients weighing at least 80 and less than 90 kg, double-dose SAP was significantly associated with a lower SSI rate (aOR, 0.76; 95% CI, 0.61-0.97; *P* = .02). In contrast, double-dose SAP was not associated with lower SSI rate in the 10 324 patients weighing at least 90 and less than 100 kg (aOR, 1.12; 95% CI, 0.87-1.47; *P* = .37), nor among the 8099 patients weighing at least 100 and less than 120 kg (aOR, 0.99; 95% CI, 0.76-1.30; *P* = .96), nor among the 2594 patients weighing at least 120 kg (aOR, 0.65; 95% CI, 0.42-1.01; *P* = .06) (Table 4).

**Table 4.** Results of Adjusted Mixed-Effects Logistic Models, Stratified by Weight Category

Weight category, kg	Patients, No.^a^	aOR (95% CI)^b^	*P* value
80 to <90	15 664	0.76 (0.61-0.97)	.02
90 to <100	9640	1.12 (0.87-1.47)	.37
100 to <120	7522	0.99 (0.76-1.30)	.96
≥120	2388	0.65 (0.42-1.01)	.06

Abbreviation: aOR, adjusted odds ratio.

^a^ Complete cases only.

^b^ Estimates are provided for the association of double dose cefuroxime (3.0 g) with surgical site infection; ie, reference category is single-dose cefuroxime (1.5 g).

Next, we stratified for wound contamination class. Double-dose SAP was significantly associated with a lower SSI rate within the 1946 patients (5.2%) with contaminated wounds (aOR, 0.49; 95% CI, 0.30-0.84; *P* = .008) but not among the 25 680 patients (68.2%) with clean wounds (aOR, 0.92; 95% CI, 0.76-1.12; *P* = .44), nor among the 10 014 patients (26.6%) patients with clean-contaminated wounds (aOR, 0.90; 95% CI, 0.73-1.12; *P* = .37) (eTable 1 in Supplement 1).

Supplementary analyses of the complete data set with the outcome being complex SSI (deep wound infection and organ space infection) as well as wound infections (superficial and deep) yielded similar results as the main analysis (eTable 2 in Supplement 1). An additional analysis comparing the 10 264 patients weighing at least 80 kg in the database receiving 2 g of cefazoline with the 1073 patients receiving 3.0 g of cefazoline also showed similar results (eTable 3 in Supplement 1). Results of adjusted generalized logistic models, stratified by surgical procedure, are shown in eTable 4 in Supplement 1.

In a subgroup analysis, we excluded surgical procedures with implant that led to a second follow-up after 1 year (cardiac surgery as well as hip/knee implant surgery). In this fully adjusted model of the remaining 15 809 patients and complete records, cefuroxime double dose was significantly associated with a lower risk of SSI (aOR, 0.83; 95% CI, 0.69-0.99; *P* = .04). When comparing included cases with those with no follow-up, we noted minor differences in several characteristics (eTable 5 in Supplement 1). None of these differences suggested a substantial bias resulting from the exclusion of patients without complete follow up. Apart from the lost to follow up, the number of missing baseline covariates was 185 (0.6%) for ASA score and 2189 (5.8%) for BMI.

## Discussion

### Principal Findings

The results of this real-life cohort study show an overall unchanged SSI risk when SAP was administered as a double dose. In multivariable models, we found significant interactions with both weight categories and wound contamination classes. In the weight category–stratified models, SSI rates were 20% lower with the higher dose for patients weighing at least 80 and less than 90 kg, but significant differences were not observed in any of the higher weight categories. Second, in the models stratified by wound contamination class, SSI rates were 50% lower in patients with contaminated wounds but not with clean or clean-contaminated wounds.

Regarding the decreased risk in surgical procedures with the contaminated wound class, a previous meta-analysis^22^ identified a 46% lower SSI rate for certain intra-abdominal surgical procedures in which multiple SAP doses were administered vs a single dose. Therefore, the lower SSI rate in this wound contamination class category with the double dose may reflect the higher single-dose SAP or even a need for therapeutic (or at least prolonged) rather than single-dose prophylactic antimicrobial treatment. Of note, a recent meta-analysis^23^ found no evidence of benefit for an overall postoperative continuation of SAP. Our analysis was also not designed to answer whether single-dose or repeated SAP administration for contaminated wound surgery were associated with a differential SSI rate.

The interpretation of the lower rate among patients weighing 80 to 90 kg is more complicated and may simply represent a spurious finding. However, a hypothesis for the significantly lower rate could be that this weight category benefits from the higher dose^24^ without being overridden by the higher SSI risk associated with increased weight.^4,8,17,25^ In addition, it has been shown that cefazolin tissue concentration is reduced with increasing weight, and therefore, even higher doses may be required for individuals weighing more than 90 kg,^26^ while mean serum concentrations remained similar independent of the weight category.^12^

Our primary exposure variable was single- vs double-dose SAP administration. However, our data show that factors other than timing of SAP administration were significantly associated with SSI risk. Increased weight, higher ASA score, and unplanned procedures were strongly associated with an increased risk.^17^

### Internal and External Validity

We believe the internal validity or our study to be excellent, as hospitals throughout Switzerland participated, including smaller institutions (<200 beds) and large centers (>500 beds). The multilevel analysis with clustering at the intervention level allowed us to control for potential variation in SSI rate between different surgical procedures. In addition, we adjusted for hospital size and individual factors (age, ASA score, duration of surgery) that might have been a possible source of bias. Uniform SAP recommendations for Switzerland were introduced in 2015. Antimicrobial resistance rates (eg, methicillin-resistant *Staphylococcus aureus*, extended spectrum β-lactamase) are low throughout the country, not requiring broader empirical SAP coverage. Therefore, it is unlikely that centers varied their SAP protocols significantly according to their local epidemiology.

Strengths of our study were the large sample size, standardized evaluation of SSI cases by dedicated nurses and physicians, postdischarge surveillance at 30 days (or 1 year for implant surgery) and a less than 9% loss to follow-up. In addition, our study involved routine on-site monitoring of the data collection quality and a multilevel model that allowed adjustment for different surgical procedures.

Concerning external validity, the analysis of large prospective registries may be the ideal source for generating high-quality scientific data.^27^ Our results did not confirm a preliminary study that suggested an approximately 4-fold lower SSI risk among patients weighing at least 80 kg who received a double cefuroxime dose.^16^ The 4-fold lower rate is unlikely to be physiological, and therefore, previous studies may not have corrected for significant, unrecognized bias.

### Clinical Implications

Our results suggest that general routine administration of a double SAP dose in patients weighing at least 80 kg has no general additional benefit. The observed signal in the weight category of 80 to 90 kg and the lower rate in patients with contaminated wounds and in surgical procedures without implants must be further confirmed. Given its minor toxic effects^8^ and the significant association in 2 stratified analysis, application of double-dose cefuroxime SAP in patients weighing at least 80 kg merits further considerations.

### Research Implications

To definitively answer the question of whether a dose increase may lower SSI rate in patients weighing at least 80 kg and for this strategy to become standard practice, randomized clinical trials are needed. In consideration of the very large sample size of the present cohort study, this will be hard to achieve.

### Limitations

This study has limitations. The main limitation was that variables were predefined by the SSI surveillance program. Important patient comorbidities and characteristics, such as diabetes, smoking, nutritional status, intraoperative temperature, oxygen measurements, and continued antimicrobial prophylaxis, were not available.

As this was a real-life cohort study, there may have been confounding by indication, which could have led to underestimation of a significant association of double-dose SAP. The results may have been biased by including procedures with implants and a 1-year follow up. When excluding these patients, double-dose cefuroxime was significantly associated with a lower SSI rate. These patients may be more prone to infection independent of the exposure to different doses of surgical antimicrobial prophylaxis.

We also lacked information on individual surgeons as well as on individual decisions regarding when single or double doses were administered. In addition, there were no serum or tissue cefuroxime levels available. We did not assess toxic effects or antimicrobial agent serum concentrations that were associated with the 2 different doses. As routine susceptibility of microorganisms was not available, we were not able to assess the association between the dose of cefuroxime SAP and cefuroxime-susceptibility of microorganisms identified.

## Conclusions

In this study, double-dose cefuroxime SAP in patients weighing at least 80 kg was not consistently associated with a lower SSI rate. The lower SSI rate within the weight category of 80 to less than 90 kg, for contaminated wound class, and for surgical procedures without implants merits further investigation.

## Notes

Supplement 1. **eTable 1.** Results of Adjusted Mixed-Effects Logistic Models, Stratified by Wound Contamination Class **eTable 2.** Fully Adjusted Mixed-Effects Logistic Regression Models With Surgical Site Infection as the Dependent Variable, by Tissue Level of Infection **eTable 3.** Fully Adjusted Mixed-Effects Logistic Regression Models With Surgical Site Infection as the Dependent Variable for the Cefazolin Double-Dose Model **eTable 4.** Fully Adjusted Generalized Linear Models With Surgical Site Infection as the Dependent Variable, Stratified by Surgical Procedure Type **eTable 5.** Missing Data Analysis: Patients With/Without Follow-up

Supplement 2.
Nonauthor Collaborators
