# Supplementary material for: Association Between Antimicrobial Prophylaxis With Double-Dose Cefuroxime and Surgical Site Infections in Patients Weighing 80 kg or More
Source: JAMA Netw Open. 2021 Dec 15;4(12):e2138926. doi: 10.1001/jamanetworkopen.2021.38926 (PMC8674749; doi:10.1001/jamanetworkopen.2021.38926)
Supplement: Supplement 1. — eTable 1. Results of Adjusted Mixed-Effects Logistic Models, Stratified by Wound Contamination Class eTable 2. Fully Adjusted Mixed-Effects Logistic Regression Models With Surgical Site Infection as the Dependent Variable, by Tissue Level of Infection eTable 3. Fully Adjusted Mixed-Effects Logistic Regression Models With Surgical Site Infection as the Dependent Variable for the Cefazolin Double-Dose Model eTable 4. Fully Adjusted Generalized Linear Models With Surgical Site Infection as the Dependent Variable, Stratified by Surgical Procedure Type eTable 5. Missing Data Analysis: Patients With/Without Follow-up [file jamanetwopen-e2138926-s001.pdf]

Supplemental Online Content

Sommerstein R, Atkinson A, Kuster SP, et al; Swissnoso Network. Association between antimicrobial prophylaxis with double-dose cefuroxime and surgical site infections in patients weighing 80 kg or more. *JAMA Netw Open.* 2021;4(12):e2138926. doi:10.1001/jamanetworkopen.2021.38926

eTable 1. Results of Adjusted Mixed-Effects Logistic Models, Stratified by Wound Contamination Class
eTable 2. Fully Adjusted Mixed-Effects Logistic Regression Models With Surgical Site Infection as the Dependent Variable, by Tissue Level of Infection
eTable 3. Fully Adjusted Mixed-Effects Logistic Regression Models With Surgical Site Infection as the Dependent Variable for the Cefazolin Double-Dose Model
eTable 4. Fully Adjusted Generalized Linear Models With Surgical Site Infection as the Dependent Variable, Stratified by Surgical Procedure Type
eTable 5. Missing Data Analysis: Patients With/Without Follow-up

This supplemental material has been provided by the authors to give readers additional information about their work.

© 2021 Sommerstein R et al. *JAMA Network Open.*

eTable 1. Results of Adjusted Mixed-Effects Logistic Models, Stratified by Wound Contamination Class

Estimates are provided for the association of double dose cefuroxime (3gr) with surgical site infection (reference = single dose cefuroxime, 1.5gr).

Wound contamination class	n*	aOR and 95% CI	p-value
Clean	23,824	0.92 (0.76-1.12)	0.439
Clean-contaminated	9,546	0.90 (0.73-1.12)	0.372
Contaminated	1,868	0.49 (0.30-0.84)	0.008

*complete cases only

CI	Confidence interval
aOR	Adjusted odds ratio

© 2021 Sommerstein R et al. *JAMA Network Open.*

eTable 2. Fully Adjusted Mixed-Effects Logistic Regression Models With Surgical Site Infection as the Dependent Variable, by Tissue Level of Infection

Procedure type was added as random effect. Only complete cases (35,268/37,640)

Tissue level of Surgical site infection	aOR	LL 95% CI	UL 95% CI	p-value
Superficial	0.93	0.73	1.19	0.599
Deep	0.96	0.68	1.36	0.830
Organ space	0.86	0.72	1.03	0.110
Wound infection (superficial/deep)	0.91	0.75	1.10	0.332
Complex (deep wound and organ space)	0.88	0.75	1.04	0.132

© 2021 Sommerstein R et al. *JAMA Network Open.*

eTable 3. Fully Adjusted Mixed-Effects Logistic Regression Models With Surgical Site Infection as the Dependent Variable for the Cefazolin Double-Dose Model

Procedure type was added as random effect. Only complete cases (9,992/10,491)

Variable	aOR	LL 95% CI	UL 95% CI	p-value
Cefazoline double dose (Ref=single dose)	0.93	0.64	1.36	0.726
BMI (per 1 m/kg^2)	1.07	1.04	1.09	<0.001
Age (per year)	1.02	1.01	1.03	0.002
Male Sex (Ref=female)	1.49	1.08	2.06	0.014
ASA Score 3-5 (Ref = ASA 1 / 2)	1.52	1.14	2.02	0.004
Wound contamination class (Ref = clean)				
Clean-contaminated	2.02	0.87	4.69	0.101
Contaminated	2.20	0.88	5.47	0.091
Elective surgery (Ref=No)	0.55	0.40	0.76	<0.001
Timing of SAP before incision (per 30min)	0.88	0.75	1.03	0.106
Duration exceeding standard time (Ref = yes)	1.02	0.78	1.34	0.864
Year (Ref= 2015)				
2016	0.84	0.51	1.38	0.489
2017	1.01	0.62	1.65	0.961
2018	0.73	0.43	1.23	0.238
2019	0.85	0.49	1.48	0.558
Hospital Size (Ref= <200 beds)				
200-499 beds	1.45	1.07	1.96	0.016
500+ beds	1.82	1.33	2.49	<0.001

Abbreviations

ASA	American Society of Anesthesiologists
CI	Confidence interval
LL	Lower limit
UL	Upper limit
aOR	Adjusted odds ratio

© 2021 Sommerstein R et al. *JAMA Network Open.*

eTable 4. Fully Adjusted Generalized Linear Models With Surgical Site Infection as the Dependent Variable, Stratified by Surgical Procedure Type

Estimates are provided for the association of double dose cefuroxime (3gr) with surgical site infection (reference = single dose cefuroxime, 1.5gr).

Surgical procedure	Complete cases	aOR (95% CI)	p-value
Gastric bypass	2,948	0.72 (0.44-1.23)	0.227
Colon	3,007	0.80 (0.64-1.01)	0.066
Laminectomy	1,820	0.49 (0.17-1.34)	0.171
Cholecystecomy	2,393	1.24 (0.57-2.65)	0.569
Cardiac surgery	3,486	1.15 (0.83-1.62)	0.415
Total knee prothesis	7,923	0.81 (0.49-1.29)	0.383
Total hip prothesis	8,047	0.87 (0.61-1.23)	0.432
Hernia repair	2,677	0.74 (0.26-1.95)	0.561
C-section	2,948	0.72 (0.44-1.23)	0.227

Abbreviations

aOR Adjusted odds ratio

CI Confidence interval

© 2021 Sommerstein R et al. *JAMA Network Open.*

eTable 5. Missing Data Analysis: Patients With/Without Follow-up

	Single dose (1500mg)	Double dose (3000mg)	p
n	37640	3436	
Age (median [IQR])	61.9 [49.9, 71.0]	61.5 [50.6, 70.9]	0.851
Sex = m (%)	22625 (60.1)	1988 (57.9)	0.01
Weight category (%)			0.241
80-90 kg	16605 (44.1)	1460 (42.5)	
90-100 kg	10342 (27.5)	980 (28.5)	
100-120 kg	8099 (21.5)	766 (22.3)	
>120 kg	2594 (6.9)	230 (6.7)	
ASA scores (%)			0.019
1, 2	24209 (64.3)	2149 (62.5)	
3, 4, 5	13246 (35.2)	1261 (36.7)	
NA	185 (0.5)	26 (0.8)	
Intervention Type (%)			<0.001
Total Knee Prosthesis	8718 (23.2)	1066 (31.0)	
Total Hip Prosthesis	8973 (23.8)	817 (23.8)	
Cardiac surgery	3529 (9.4)	375 (10.9)	
Colon surgery	3266 (8.7)	162 (4.7)	
Hernia repair	2752 (7.3)	175 (5.1)	
Cesarean Section	3038 (8.1)	231 (6.7)	
Cholecystectomy	2413 (6.4)	218 (6.3)	
Laminectomy	1840 (4.9)	107 (3.1)	
Gastric bypass surgery	3111 (8.3)	285 (8.3)	
Wound contamination class (%)			<0.001
I. clean	25680 (68.2)	2522 (73.4)	
II. clean-contaminated	10014 (26.6)	767 (22.3)	
III. contaminated	1946 (5.2)	147 (4.3)	
Elective surgery = yes (%)	33730 (89.6)	3113 (90.6)	0.073
SAP application prior incision in minutes (median [IQR])	-38 [-50, -26]	-37 [-50, -27]	0.041
Surgery exceeding standard time = yes (%)	6593 (17.5)	530 (15.4)	0.002
Year (%)			0.056
2015	2021 (5.4)	198 (5.8)	
2016	10001 (26.6)	882 (25.7)	
2017	11792 (31.3)	1139 (33.1)	
2018	10180 (27.0)	922 (26.8)	
2019	3646 (9.7)	295 (8.6)	
Hospital size (beds, %)			<0.001
<200	22377 (59.5)	1965 (57.2)	
200-499	10820 (28.7)	1133 (33.0)	
500+	4443 (11.8)	338 (9.8)	

© 2021 Sommerstein R et al. *JAMA Network Open.*
